# Accuracy of the SD BIOLINE Dengue Duo for rapid point-of-care diagnosis of dengue

**DOI:** 10.1371/journal.pone.0213301

**Published:** 2019-03-06

**Authors:** Mariana Kikuti, Jaqueline S. Cruz, Moreno S. Rodrigues, Aline S. Tavares, Igor A. D. Paploski, Monaise M. O. Silva, Perla M. Santana, Laura B. Tauro, Greice A. O. F. Silva, Gúbio S. Campos, Josélio M. G. Araújo, Uriel Kitron, Mitermayer G. Reis, Guilherme S. Ribeiro

**Affiliations:** 1 Instituto Gonçalo Moniz, Fundação Oswaldo Cruz, Salvador, Bahia, Brazil; 2 Instituto de Saúde Coletiva, Universidade Federal da Bahia, Salvador, Bahia, Brazil; 3 Department of Veterinary Population Medicine, University of Minnesota, Saint Paul, Minnesota, United States of America; 4 Instituto Nacional de Medicina Tropical, CONICET, Puerto Iguazu, Misiones, Argentina; 5 Instituto de Ciências da Saúde, Universidade Federal da Bahia, Salvador, Bahia, Brazil; 6 Departamento de Microbiologia e Parasitologia, Universidade Federal do Rio Grande do Norte, Natal, Rio Grande do Norte, Brazil; 7 Department of Environmental Science, Emory University, Atlanta, Georgia, United States of America; 8 Department of Epidemiology of Microbial Diseases, Yale School of Public Health, New Haven, Connecticut, United States of America; 9 Faculdade de Medicina da Bahia, Universidade Federal da Bahia, Salvador, Bahia, Brazil; Hong Kong Institute for the Humanities and Social Sciences, HONG KONG

## Abstract

**Background:**

Rapid diagnosis tests (RDTs) are easy to carry out, provide fast results, and could potentially guide medical treatment decisions. We investigated the performance of a commercially available RDT, which simultaneously detects the non-structural 1 (NS1) dengue virus (DENV) antigen, and IgM and IgG DENV antibodies, using representative serum samples from individuals in a dengue endemic area in Salvador, Brazil.

**Methodology/Principal findings:**

We evaluated the accuracy of the SD BIOLINE Dengue Duo RDT (Abbott, Santa Clara, USA; former Alere Inc, Waltham, USA) in a random collection of sera. Samples included acute-phase sera from 246 laboratory-confirmed dengue cases and 108 non-dengue febrile patients enrolled in a surveillance study for dengue detection, 73 healthy controls living in the same surveillance community, and 73 blood donors. RDT accuracy was blindly assessed based on the combined results for the NS1 and the IgM test components. The RDT sensitivity was 46.8% (38.6% for the NS1 component and 13.8% for the IgM component). Sensitivity was greater for samples obtained from patients with secondary DENV infections (49.8%) compared to primary infections (31.1%) (P: 0.02) and was also influenced by the result in the confirmatory dengue diagnostic test, ranging from 39.7% for samples of cases confirmed by IgM-ELISA seroconversion between paired samples to 90.4% for samples of cases confirmed by a positive NS1-ELISA. The RDT specificity was 94.4% for non-dengue febrile patients, 87.7% for the community healthy controls, and 95.9% for the blood donors.

**Conclusions/Significance:**

The SD BIOLINE Dengue Duo RDT showed good specificities, but low sensitivity, suggesting that it may be more useful to rule in than to rule out a dengue diagnosis in dengue endemic regions.

## Introduction

Dengue virus (DENV) is estimated to infect up to 390 million people each year, of which 96 million develop clinical disease [1]. Symptomatic DENV infections may cause a wide spectrum of acute clinical manifestations, from self-limited, mild, and non-specific symptoms, such as fever, myalgia, and headache, to severe disease evolving with plasma leakage, hemorrhage, and eventually death [2]. Thus, early diagnosis is critical to guide timely fluid replacement that can prevent disease complications and death [3].

Several laboratory exams for dengue diagnosis are commercially available, including molecular tests for detection of viral DENV RNA by reverse-transcription polymerase chain reaction (RT-PCR), enzyme-linked immunosorbent assays (ELISAs) for detection of non-structural 1 (NS1) DENV protein during the viremic phase and detection of DENV IgM antibodies after the viremic phase, among many others. The choice of which diagnostic method to be used needs to consider the timing of blood sample collection during the disease course, as well as other advantages and limitations of each technique, such as accuracy, cost, and the need for specialized personnel and equipment [4]. Molecular tests and ELISAs are typically time-consuming and require laboratory infrastructure not commonly found in outpatient health units. Thus, they are of limited use for rapid evaluation of patients suspected of dengue and often do not help guiding medical treatment in such settings.

Rapid tests, which are easy to implement and give fast results, could potentially assist medical diagnosis and treatment decisions. Yet, they generally present lower sensitivity and specificity than the other recommended tests, and, therefore, their results have to be interpreted with caution [2]. There is a wide range of commercially available rapid diagnostic tests (RDTs) for dengue that focus primarily on the detection of IgM or IgG antibodies, detection of the NS1 DENV antigen, or in a combination of them. Although previous studies have evaluated the accuracy of these tests, few assessed the performance of these tests among outpatients from dengue endemic areas presenting with acute febrile illness, a population who might benefit greatly from early dengue diagnosis.

Herein, we present results of a retrospective evaluation of the diagnostic performance of a commercial RDT among febrile outpatients from Salvador, Brazil, a city with endemic DENV transmission. Among the many dengue RDTs commercially available, we selected to evaluate the SD BIOLINE Dengue Duo test (Abbott, Santa Clara, USA; former Alere Inc, Waltham, USA), because of its capability to detect simultaneously the NS1 antigen, and IgM and IgG DENV antibodies. Furthermore, it has been used in some Brazilian state public health laboratories during dengue epidemics.

## Methods

### Serum sample groups

**Fig 1:**
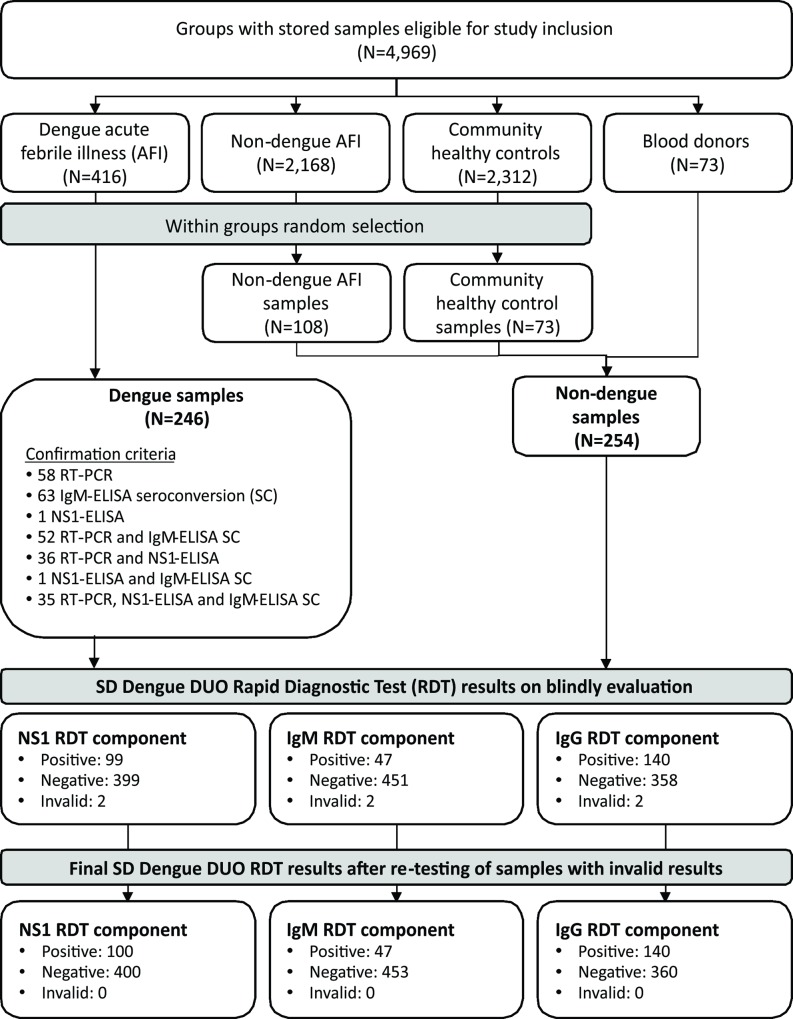
Serum sample selection and SD BIOLINE Dengue Duo testing.

To evaluate the RDT accuracy we used 500 representative serum samples collected, processed, and stored following good clinical practices, comprising the following four well characterized groups: i) dengue cases confirmed by laboratory reference tests during a surveillance study for acute febrile illness (AFI) (N: 246 samples); ii) non-dengue AFI controls enrolled in the same surveillance study (N: 108 samples); iii) presumably healthy individuals living in the same community where the surveillance study was conducted (healthy community controls) (N: 73 samples); and iv) blood donors (N: 73 samples) (Fig 1). All the 500 serum samples undergoing RDT evaluation were stored at -20°C until use and had never been thawed before. Sample size was determined based on an average RDT sensitivity and specificity of 85% and 95%, respectively [5–8], with a minimum precision of 5% and 95% confidence interval. Next, we describe the procedures for obtaining and selecting the serum samples from each of the evaluated groups.

### Samples from dengue and non-dengue AFI patients

Between January 2009 and December 2011, we conducted a community-based enhanced surveillance study aimed to detect DENV infections among AFI patients [9,10]. The AFI surveillance was performed in a public outpatient health unit, located in the Pau da Lima community, Salvador, Brazil. This area has endemic dengue transmission, with an estimated incidence of 21 and 70 cases per 10,000 inhabitants in 2009 and 2010, respectively [10]. The surveillance team prospectively enrolled patients fulfilling the following inclusion criteria: age of five years or more, reported fever or measured axillary temperature ≥37.8°C of up to 21 days of duration, and residence in the community. Enrollment was performed Monday to Friday, from 07h30 to 16h00.

Participants’ blood samples were collected at enrollment (acute-phase sample) and ≥ 15 days post enrollment (convalescent-phase sample), maintained refrigerated at 2–8°C and transported on the day of collection to our laboratory at the Gonçalo Moniz Institute for centrifugation. Sera were then stored in aliquots at -20°C and -70°C for serological and molecular testing, respectively. We tested the acute-phase sera samples with DENV NS1- and IgM-ELISA, and the convalescent-phase sera with DENV IgM-ELISA (Abbott, Santa Clara, USA; former Panbio Diagnostics, Brisbane, Australia) according to manufacturer’s instructions. Patients with a positive result in either of the serological tests and patients enrolled between February and July 2010, a period when an outbreak of DENV was ongoing in Salvador, had their acute-phase sample also tested by RT-PCR [11].

We defined as a dengue confirmed case the AFI patients who presented a positive NS1-ELISA, an IgM-ELISA seroconversion (a negative result in the acute-phase IgM-ELISA followed by a positive result in the convalescent-phase IgM-ELISA), or a positive DENV RT-PCR. Patients presenting only an IgM-ELISA positive result in the acute-phase sample were not considered as confirmed dengue cases, neither as non-dengue AFI cases because we could not accurately differentiate those presenting an acute infection from those who had a previous, recent infection and still maintained DENV IgM antibodies. A non-dengue case was defined as an AFI patient without any positive laboratory result in the performed tests. Dengue confirmed cases also had their acute-phase serum samples tested by IgG-ELISA (Abbott, Santa Clara, USA; former Panbio Diagnostics, Brisbane, Australia) to determine the type of infection (primary, defined by a negative result in the IgG-ELISA; versus secondary, defined by a positive result in the IgG-ELISA).

During the surveillance period, 4,520 AFI patients were enrolled; 571 (12.6%) were classified as confirmed dengue cases and 3,380 (74.8%) as non-dengue AFI patients (an additional 569 (12.6%) patients tested positive for DENV IgM-ELISA in the acute-phase serum). From the 416 (72.9%) dengue and 2,168 (64.1%) non-dengue AFI patients with available serum sample, we randomly selected for this RDT evaluation 246 dengue cases and 108 non-dengue cases.

### Samples from healthy community controls

Serum samples from participants of a household-based random survey, conducted among 5 or more years of age residents of Pau da Lima, between January and June 2010, were included in the RDT evaluation study representing a group of presumably healthy community controls from a dengue endemic region. We randomly selected 73 serum samples from the 2,312 survey participants for testing by the SD BIOLINE Dengue Duo RDT.

### Samples from blood donors

Lastly, serum samples from 73 volunteer blood donors from Salvador metropolitan area were included as an additional group of negative control. Blood donors’ samples were prospectively collected in December 2013, after the donators underwent the health triage, which included age (16 to 69 years), weight (> 50kg), and health restrictions (viral hepatitis, HIV infection, pregnancy, breastfeeding, risky behavior for blood borne infectious diseases, and fever in the 15 days prior to blood donation).

### Blinding

To blind the RDT readers, the 500 selected samples were shuffled and re-identified with sequential numbers. Readers had no access to the dataset containing the key code of the samples and did not participate in the re-identification process. Two independent blinded members of the study team entered the RDT results into a dataset, which were then validated to assure data quality. The complete dataset containing all diagnostic results for each patients’ sample were only available for data analysis after all laboratory tests were completed and inserted in the database (S1 Dataset).

### RDT evaluation

The SD BIOLINE Dengue Duo RDT were stored in a closed room with air conditioning before use. Testing was performed according to the manufacturer’s instruction. Briefly, we added 100 μL of serum in the NS1 component, and 10 μL of serum and 4 drops of the buffer solution in the IgM and IgG component. Three independent blinded readers, previously trained by a manufacturer’s representative, interpreted the RDT results in a bright area within 15–20 minutes. Each component of the RDT test was considered positive if it showed two positive bands (the band of the tested component and the internal control band), negative if it presented only one band (internal control band), and invalid if the internal control band was absent. The three readers independently recorded each test component reading result. The most frequent reading result for each test component was considered the final test component result. Each sample was tested once, unless the RDT presented an invalid result. If so, the test was repeated according to the same protocol, and the most frequent result between the tree readers in the repetition test was considered final.

We also evaluated test-to-test reproducibility by randomly selecting 50 samples (10%) and repeating the testing protocol. In addition, we randomly selected 30 patients of all laboratory-confirmed dengue cases who had a negative IgM result in the RDT and who had a convalescent-phase serum sample available, and tested the convalescent-phase sample with the RDT.

To investigate false positive results in the RDT, we performed RT-PCR [11] in serum samples (stored at -70°C) of all non-dengue AFI controls who had a positive result in either the NS1- or IgM-RDT component to assure they had been correctly classified as non-dengue cases. Likewise, to assure that the community and blood donor control groups did not include an asymptomatic dengue infection during the time of sample collection, we performed NS1- and IgM-ELISAs on their -20°C serum samples. RT-PCR was not performed because these control groups did not have serum samples stored at -70°C.

### Statistical analysis

We used frequencies and medians plus interquartile ranges (IQR) to describe demographics and clinical characteristics of the participants from each of the serum sample groups included in this study and compared them using chi-square or Kruskal-Wallis equality-of-populations tests. We assessed sensitivity and specificity for the IgM and the NS1 test components, both separately and in combination, and considered the combined IgM and NS1 reading records as the final RDT result. The accuracy of the IgG RDT component was not evaluated because prior studies have shown that the prevalence of IgG antibodies in regions endemic for dengue can reach 97%, making detection of DENV IgG of limited use for diagnosis in such settings [12,13].

RDT sensitivity was also calculated by type of dengue infection (primary or secondary), DENV serotype, number of days of symptoms (0–2, 3–4, and ≥5), and laboratory confirmation criteria, and differences in the sensitivity according to these patients’ characteristics were assessed by chi-square or chi-square for trend test. RDT specificity was assessed for each control group separately. Additionally, sensitivity and specificity for the NS1 and IgM RDT components were also assessed against their analogous reference test (NS1- and IgM-ELISAs, respectively). Confidence intervals at 95% (95% CI) were calculated for all accuracy measurements. Inter-operator agreement between RDT readers and test-to-test reproducibility were calculated using global agreement and Kappa. Invalid results on the RDT or equivocal results on the ELISAs were excluded from the analysis. All statistical analysis were done using Stata 14 [14].

### Ethics statement

This study was designed, conducted, and reported according to the Evaluation of Diagnostic Tests for Dengue Guidelines [15] and the STARD (Standards for Reporting of Diagnostic Accuracy) Guideline (S1 File). The study was approved by the Research Ethics Committee at the Gonçalo Moniz Institute, Oswaldo Cruz Foundation. All adult AFI patients and control subjects provided written informed consent. Participants <18 years old who were able to read provided written assent following written consent from their parent or guardian. All study data were anonymized before analysis.

## Results

### Characteristics of the study participants

Of the 246 laboratory-confirmed dengue patients whose samples were selected for this study, 181 (73.6%) were RT-PCR positive, 72 (29.3%) were NS1-ELISA positive, and 151 (61.4%) presented IgM-ELISA seroconversion between the acute- and convalescent-phase samples. More than one laboratory criteria for diagnosis occurred for 124 (50.4%) of the dengue cases. Of the 246 dengue confirmed cases, 199 (80.1%) had a secondary infection.

Dengue and non-dengue AFI patients whose samples were selected for this study were similar regarding sex (42.3% and 44.4% were male, respectively; P = 0.70) and age distribution (median in years [IQR]: 18 [10–31] and 17 [10–30], respectively; P = 0.23) (Table 1). They also reported similar frequencies of headache (>80%), prostration (>75%) and myalgia (>70%). However, the following manifestations were more frequent among dengue cases than among non-dengue AFI controls: retro-orbital pain (59.8% vs. 42.6%; P = 0.001), arthralgia (46.3% vs. 32.4%; P = 0.01), and rash (21.5% vs. 12.0%; P = 0.02).

**Table 1:** Characteristics of laboratory-confirmed dengue cases and of non-dengue acute febrile illness cases whose sera were included in the panel for the SD BIOLINE Dengue Duo rapid test evaluation.

Characteristics			Dengue AFI (n = 246)	Non-dengue AFI (n = 108)	*p*-value^a^
			N (%) or median (IQR)		
Age in years			18 (10–31)	17 (10–30)	0.23
Male sex			104 (42.3)	48 (44.4)	0.73
Days of symptoms at enrollment			2 (2–4)	3 (1–4)	0.42
Clinical manifestations					
	Headache		214 (87.0)	91 (84.3)	0.50
	Prostration		195 (79.3)	82 (75.9)	0.48
	Myalgia		187 (76.0)	77 (71.3)	0.35
	Retro-orbital pain		147 (59.8)	46 (42.6)	0.003
	Arthralgia		114 (46.3)	35 (32.4)	0.02
	Rash		53 (21.5)	13 (12.0)	0.04
	Vomiting		74 (30.1)	30 (27.8)	0.71
Clinical diagnoses recorded at medical chart					
	None		144 (58.5)	56 (51.9)	0.25
	At least one clinical diagnosis ^b^		102 (41.5)	52 (48.1)	
		Dengue	39 (39.4)	2 (3.9)	<0.001
		Upper respiratory tract infections	16 (16.2)	18 (34.6)	0.01
		Undetermined viral disease	28 (28.3)	7 (13.5)	0.04
		Gastrointestinal tract infections	4 (4.0)	2 (3.9)	1.00
		Urinary tract infections	2 (2.0)	1 (1.9)	1.00
		Pneumoniae	1 (1.0)	1 (1.9)	1.00
		Sinusitis	0 (0)	3 (5.8)	0.04
		Leptospirosis	3 (3.0)	2 (3.9)	1.00
		Tonsillitis	10 (10.1)	12 (23.1)	0.05

AFI, Acute febrile illness.

^a^ Wilcoxon (Mann-Whitney) rank-sum test or Fisher's exact test.

^b^ Some patients had more than one clinical diagnosis recorded in their medical chart.

Less than half of the dengue and non-dengue patients had a clinical suspicion recorded in their medical chart (Table 1). Among those with a recorded clinical suspicion, dengue was suspected for 39.4% of the dengue confirmed cases and for 3.9% of the non-dengue AFI controls (P<0.001). The most common suspected diagnosis for the non-dengue AFI controls were upper respiratory tract infections (34.6%).

The community health controls comprised 37.0% males, with a median age of 25 [14–37] years, and the blood donors comprised 64.4% males, with a median age of 33 [27–41] years (P<0.001 for sex and age comparison between the four groups, respectively).

### Overall RDT sensitivity

Of the 500 tested samples, only 2 (0.4%) needed to be retested to achieve a valid result (both in the NS1 and IgM/IgG components). Sensitivities for the NS1 and IgM components of the RDT were 38.6% (95% CI: 32.5–45.0) and 13.8% (95% CI: 3.9–18.8), respectively (Table 2). Considering a positive result in either the NS1 or the IgM component of the RDT, the combined sensitivity of the RDT was 46.8% (95% CI: 40.4–53.2). The IgG RDT component was positive for 64 (26.0%) of the 246 dengue cases, all of them with a secondary infection defined by a positive IgG-ELISA in the acute-phase sample (32.2% of the 199 secondary dengue infections detected by the IgG-ELISA).

**Table 2:** Sensitivity of NS1 and IgM SD BIOLINE Dengue Duo rapid diagnostic test (RDT) components according to type of infection, infecting serotype, number of days post-onset of symptoms, and reference test confirmation criteria.

Characteristics of laboratory-confirmed dengue patients			No. tested samples	NS1 or IgM RDT component		NS1 RDT component		IgM RDT component	
				No. positive samples	Sensitivity (95% CI)	No. positive samples	Sensitivity (95% CI)	No. positive samples	Sensitivity (95% CI)
Overall			246	115	46.8 (40.4–53.2)	95	38.6 (32.5–45.0)	34	13.8 (9.8–18.8)
Type of infection ^a^									
	Primary		45	14	31.1 (18.2–46.7)	12	26.7 (14.6–41.9)	2	4.4 (0.5–15.2)
	Secondary		199	99	49.8 (42.6–56.9)	81	40.7 (33.8–47.9)	31	15.6 (10.8–21.4)
Serotype									
	DENV1		18	11	61.1 (35.8–82.7)	10	55.6 (30.8–78.5)	2	11.1 (1.4–34.7)
	DENV2		113	61	54.0 (44.4–63.4)	52	46.0 (36.6–55.7)	18	15.9 (9.7–24)
	DENV4		49	34	69.4 (54.6–81.8)	30	61.2 (46.2–74.8)	7	14.3 (5.9–27.2)
Days of symptoms ^b^									
	≤4		213	102	47.9 (41.0–54.8)	89	41.8 (35.1–48.7)	25	11.7 (7.7–16.8)
		≤2	125	60	48.0 (39.0–57.1)	54	43.2 (34.4–52.4)	8	6.4 (2.8–12.2)
		3	56	23	41.1 (28.1–55.0)	19	33.9 (21.8–47.8)	7	12.5 (5.2–24.1)
		4	32	19	59.4 (40.6–76.3)	16	50.0 (31.9–68.1)	10	31.3 (16.1–50.0)
	≥5		30	11	36.7 (19.9–56.1)	5	16.7 (5.6–34.7)	8	26.7 (12.3–45.9)
Reference tests included a positive ^c^									
	RT-PCR		181	106	58.6 (51.0–65.8)	92	50.8 (43.3–58.3)	27	14.9 (10.0–21.0)
	NS1-ELISA		73	66	90.4 (81.2–96.1)	66	90.4 (81.2–96.1)	9	12.3 (5.8–22.1)
	IgM-ELISA seroconversion ^d^		151	60	39.7 (31.9–48.0)	52	34.4 (26.9–42.6)	10	6.2 (3.2–11.8)
	IgM-ELISA in the acute-phase sample ^e^		65	37	56.9 (44.0–69.2)	25	38.5 (26.7–51.4)	24	36.9 (25.3–49.8)

^a^ According to IgG-ELISA in the acute-phase sample (IgG negative: primary infection; IgG positive: secondary infection). Patients with equivocal results in the acute-phase IgG-ELISA were excluded from the analysis.

^b^ Three patients for whom the number of days of symptoms were not precisely determined were excluded from the analysis.

^c^ Patients may have fulfilled more than one confirmation criteria in the reference tests.

^d^ IgM-ELISA seroconversion between acute- and convalescent-phase sera.

^e^ A positive IgM-ELISA result in the acute-phase sample was not considered as a dengue confirmation criteria because patients with a previous, recent, but not acute infection may have a positive result. However, some of the patients confirmed by RT-PCR or NS1-ELISA had a positive IgM-ELISA result in the acute-phase sample.

### RDT sensitivity according to characteristics of the dengue confirmed cases

Sub-analyses of the RDT sensitivity revealed that specific differences among the dengue confirmed cases, such as the type of infection, the number of days with symptoms at the time of sample collection, and the laboratory result in the reference tests might influence the RDT performance (Table 2). In contrast, the overall RDT sensitivity did not significantly differ according to the infecting DENV serotype (P = 0.18).

Sensitivity for the NS1 and the IgM RDT components combined were significantly higher among secondary DENV infections than among primary DENV infections (P = 0.02) (Table 2). In addition, as expected, the NS1 RDT sensitivity decreased (P = 0.07 for trend), while the IgM RDT sensitivity improved with the increase in the number of days of symptoms at acute-phase sample collection (P<0.001 for trend). Consequently, the overall RDT sensitivity (considering both components) declined from 47.9% when the test was used in the first 4 days after disease onset to 36.7% when used in patients with ≥5 days of symptoms, but this difference was not statistically significant (P = 0.25).

The NS1 RDT component had good sensitivity (90.4%) when the dengue case had a positive NS1-ELISA as one of the confirmation criteria, but performance was poor for dengue cases confirmed by RT-PCR (50.8%) and IgM-ELISA seroconversion (34.4%) (Table 2). In contrast, the IgM RDT component presented low sensitivity regardless of the dengue confirmation criteria used and its sensitivity was low (36.9%) even for the 65 dengue confirmed cases who had a positive IgM-ELISA in the acute-phase sample.

### RDT sensitivity in a subset of convalescent-phase samples

We randomly selected 30 of the 212 dengue confirmed patients with a negative IgM-RDT result in the acute-phase serum and an available convalescent-phase serum in order to test the convalescent-phase sample by the RDT. We found that none of the 30 convalescent-phase samples tested were positive by the NS1 RDT component, but 13 (43.3%) were positive by the IgM component. Of note, the IgM-ELISA was positive in 28 (93.3%) of the 30 convalescent-phase sera samples. As 10 (33.3%) of these 30 patients had the NS1 RDT component positive in the acute-phase sample, altogether, the paired sera evaluation resulted in a RDT sensitivity of 76.7% (23 out of 30).

### RDT specificity

The combined NS1 and IgM RDT specificity were 94.4% for non-dengue AFI patients, 87.7% for community healthy controls, and 95.9% for blood donor controls (Table 3). Specificity of the NS1 component ranged from 97.3% to 98.6% (98.0% for the combined control groups) and was higher than the specificity for the IgM-component, which ranged from 90.4% to 97.3% (94.9% for the combined control groups), but the difference was not statistically significant when we considered the three control groups combined (P = 0.06).

**Table 3:** Specificity of the NS1 and IgM SD BIOLINE Dengue Duo Rapid Diagnostic Test (RDT) components according to control group.

RDT component evaluated by control group		No. tested samples	No. negative samples	Specificity % (95% CI)
Non-dengue AFI				
	NS1 RDT	108	106	98.2 (93.5–99.8)
	IgM RDT	108	104	96.3 (90.8–99.0)
	NS1 or IgM RDT	108	102	94.4 (88.3–97.9)
Community's healthy controls				
	NS1 RDT	73	71	97.3 (90.5–99.7)
	IgM RDT	73	66	90.4 (81.2–96.1)
	NS1 or IgM RDT	73	64	87.7 (77.9–94.2)
Blood donors				
	NS1 RDT	73	72	98.6 (92.6–100.0)
	IgM RDT	73	71	97.3 (90.5–99.7)
	NS1 or IgM RDT	73	70	95.9 (88.5–99.1)

Acute-phase samples of all non-dengue AFI patients with a positive result in either the NS1- or IgM-RDT component were tested by DENV RT-PCR and presented negative results. Of the 9 community control samples with positive results in the RDT, 2 were also positive when tested by IgM-ELISA and none were positive by NS1-ELISA. None of the 3 blood donor control samples with positive results in the RDT were positive by NS1- or IgM-ELISA.

### Reproducibility

Inter-operator agreement in visual interpretation of the test ranged from 96.0% to 98.8% (kappa ranging 0.87–0.96) for the NS1-RDT component, 94.4% to 97.8% (kappa 0.63–088) for the IgM-RDT, and 87.0% to 94.0% (kappa 0.67–0.86) for the IgG-RDT. Test-to-test reproducibility with 50 randomly selected samples revealed a global agreement of 94.0% (kappa = 0.81) for the NS1-RDT component, 96.0% (kappa = 0.81) for the IgM-RDT component, and 86.0% (kappa = 0.68) for the IgG component.

## Discussion

In this study, we evaluated the diagnostic performance of the commercial SD BIOLINE Dengue Duo RDT among febrile patients from an outpatient health unit in a dengue endemic region, as well as among different control groups. Although the test presented good levels of specificity (88%– 96%), its overall sensitivity (combining positivity in either the NS1 or the IgM component) was not satisfactory (46.8%). This low sensitivity was associated with a suboptimal performance of both the NS1 and the IgM components of the test in detecting dengue cases early and late in the course of the disease, respectively.

Previous evaluations of this RDT found much greater sensitivities, ranging between 75.5–97.5% [5–8,16–20]. The diversity in patients’ clinical characteristics, frequency of primary and secondary infections, infecting DENV serotypes, and reference dengue diagnostic methods used between our study and other investigations may explain the observed differences in the measured sensitivities [15,16,21]. In our study, dengue confirmed cases were outpatients with mild disease. In contrast, the majority of the other studies were conducted on hospitalized patients, which usually manifest greater disease severity and duration, consequently influencing the intensity of inflammatory and immune response [22–24]. Yet, a previous evaluation of this RDT in Cambodia, enrolling children hospitalized due to a febrile illness of no clear source, showed an overall low sensitivity (57.8%) [21]. An even lower sensitivity (30.8%) was observed during a prospective cohort study that followed school-aged children to detect febrile episodes in Colombia [21,25].

The sensitivity of this RDT according to the type of infection remains controversial. Some studies have found higher sensitivity among patients with primary DENV infections [7,26,27], while others have observed no significant difference according to the infection type [17,18,28], or have shown high sensitivity among patients with secondary DENV infections [6][8]. Those who found higher sensitivity among primary infection patients have suggested that DENV IgG antibodies, present in secondary infections, may form immune complexes with viral antigens reducing the sensitivity of the NS1 component of the RDT [18]. In addition, the lower IgM production in secondary dengue infections would reduce the sensitivity of the IgM component of the test [29]. We, however, found a higher sensitivity among secondary infections, regardless of the RDT component being evaluated. This could be partly explained because patients with secondary DENV infections boost a faster DENV-specific IgM immune response that improve the test sensitivity.

Our small number of confirmed dengue cases with ≥5 days of symptoms underpowered our capacity to detect statistical differences in the RDT sensitivity according to disease duration. However, our findings suggest that the test does not achieve sufficient performance levels even when applied later on the course of disease. First, because the sensitivity of the IgM component of the test remained low (<30%) for acute-phase samples collected from patients with ≥5 days of symptoms. Second, because when we evaluated the RDT performance on convalescent-phase samples for a subset of 30 dengue-confirmed patients with negative IgM RDT results in the acute-phase sample, we only found 13 positive samples (none positive by the NS1 component and 13 positive by the IgM component), indicating a convalescent-phase sensitivity for the RDT test of only 43.3%. Nevertheless, combining the results of the acute- and convalescent-phase samples for these 30 dengue patients, we obtained a sensitivity of 76.7%. Thus, paired sample testing may be used to confirm a dengue diagnosis when the RDT returns a negative result in the acute-phase sample.

We defined a dengue case by a combination of RT-PCR, NS1-ELISA or IgM-ELISA seroconversion to ensure high confidence that the AFI patients really had an ongoing dengue infection and to provide the diverse laboratory scenario of dengue confirmed cases. In addition, our dengue confirmed cases were randomly selected among all dengue cases prospectively identified during a surveillance study to detect dengue cases among AFI patients. Thus, the proportion of samples fulfilling the different confirmation criteria should partially represent the scenario encountered among dengue outpatients from endemic regions. This choice turned out to be correct, because we found that the accuracy of the RDT was largely influenced by the reference diagnostic method used. As an example, when considering only the samples from dengue confirmed patients with positive results in the NS1-ELISA, we found that the NS1 component of the RDT achieved a high sensitivity (90.4%). However, the overall sensitivity for the NS1 RDT component was of 38.6% because only 73 (29.7%) of our 246 confirmed dengue patients had a positive result in the NS1 ELISA. As we did in our evaluation, it is critical that studies testing the accuracy of diagnostic methods use serum samples from representative cases instead of a convenient serum sample collection.

The specificity of the RDT was high, regardless of the control group. However, the community controls presented a lower RDT specificity (87.7%) compared to other groups. This may be explained by recent dengue infections in this randomly selected representatives from a region of endemic dengue transmission [10], as IgM for dengue may persist for up to 2 or 3 months [29]. Of note, the 7 samples presenting a false positive RDT result in the IgM component were also positive in the IgM-ELISA. The visual interpretation of RDT results showed good to excellent agreements between independent readers, while test-to-test reproducibility was considered very good for the NS1 and the IgM RDT components, and good for the IgG component [30].

Some limitations of this study need to be acknowledged. First, samples were not tested for other flaviviruses, such as yellow fever (YFV) and Zika (ZIKV) viruses, which have antigenic similarities to DENV and could potentially produce serological cross-reactions. However, YFV transmission did not occur in Salvador before 2016, when an epizootic outbreak was first detected [31]. In addition, the large ZIKV epidemics that recently reached Brazil, having the northeast region as the epicenter, only started in late 2014 and, in Salvador, it peaked in May of 2015, with over 17,000 reported cases [32]. According to phylogenetic studies, ZIKV was introduced to Brazil in late 2012 or early 2013 [33,34]. Thus, although possible, it is unlikely that the subjects providing blood samples for our study have had a ZIKV infection because the AFI samples from dengue and non-dengue cases were collected between 2009 and 2011, community control samples were collected in 2010, and only the blood donor samples were obtained in 2013. Second, RT-PCR for dengue diagnosis was only systematically performed on AFI patients presenting a positive result for dengue in the NS1- or IgM-ELISA. However, to verify for potential misclassification bias among the non-dengue AFI control group, we tested their acute-phase serum sample by RT-PCR whenever the RDT gave a positive result and none of the tested samples were RT-PCR positive. Lastly, we did not include AFI patients with only a positive IgM ELISA result in the acute-phase sample among the confirmed dengue patients to ensure greater precision in our case definition, since a positive IgM ELISA in the acute-phase sample might represent either an active or a previous, recent infection. Although this approach might have influenced the RDT accuracy, when we evaluated the RDT performance among the subset of samples from patients who fulfilled our dengue case definition and who also had a positive IgM ELISA in the acute-phase sample, we still observed a suboptimal sensitivity of 56.9%.

In conclusion, this RDT showed high specificity, indicating that AFI patients with a positive result in either in the NS1 or the IgM component of the test can be confidently considered dengue cases. However, it presented a low sensitivity, and, therefore, AFI patients with a negative RDT result should not have a dengue diagnosis discarded. In this case, performing additional diagnostic tests and/or obtaining a convalescent-phase blood sample to test might help achieve a more accurate result. Prospective studies in outpatient health units, testing fresh blood instead of stored sera, are needed to evaluate the accuracy of this RDT during point-of-care use, as well as to determine its impact in proper disease clinical management.

## Supporting information

S1 File STARD (Standards for Reporting of Diagnostic Accuracy) checklist for the reporting of studies of diagnostic accuracy. (DOCX)

S1 Dataset Clinical and laboratory data of the study participants. (XLS)

## References

[1] Bhatt S, Gething PW, Brady OJ, Messina JP, Farlow AW, Moyes CL, et al The global distribution and burden of dengue. Nature. 2013;496: 504–507. 10.1038/nature12060 23563266 PMC3651993

[2] WHO. Dengue: Guidelines for diagnosis, treatment, prevention and control Geneva: World Health Organization; 2009. 23762963

[3] Teixeira MDG, Costa MDCN, Barreto ML, Mota E. Dengue and dengue hemorrhagic fever epidemics in Brazil: what research is needed based on trends, surveillance, and control experiences? Dengue e febre hemorrágica do dengue no Brasil: que tipo de pesquisas a sua tendência, vigilância e experiências. Cad Saúde Pública. 2005;21: 1307–1315. 16158135 10.1590/s0102-311x2005000500002

[4] Kao C, King C, Chao D, Wu H, Chang GJ. Laboratory diagnosis of dengue virus infection: current and future perspectives in clinical diagnosis and public health. 2005; 5–16. 15692621

[5] Wang SM, Sekaran SD. Early diagnosis of dengue infection using a commercial dengue duo rapid test kit for the detection of NS1, IGM, and IGG. Am J Trop Med Hyg. 2010;83: 690–695. 10.4269/ajtmh.2010.10-0117 20810840 PMC2929071

[6] Blacksell SD, Jarman RG, Bailey MS, Tanganuchitcharnchai A, Jenjaroen K, Gibbons R V., et al Evaluation of six commercial point-of-care tests for diagnosis of acute dengue infections: The need for combining NS1 antigen and IgM/IgG antibody detection to achieve acceptable levels of accuracy. Clin Vaccine Immunol. 2011;18: 2095–2101. 10.1128/CVI.05285-11 22012979 PMC3232692

[7] Tricou V, Vu HTT, Quynh NVN, Nguyen CV V, Tran HT, Farrar J, et al Comparison of two dengue NS1 rapid tests for sensitivity, specificity and relationship to viraemia and antibody responses. BMC Infect Dis. 2010;10: 142 10.1186/1471-2334-10-142 20509940 PMC2895602

[8] Osorio L, Ramirez M, Bonelo A, Villar L a, Parra B. Comparison of the diagnostic accuracy of commercial NS1-based diagnostic tests for early dengue infection. Virol J. BioMed Central Ltd; 2010;7: 361 10.1186/1743-422X-7-361 21134275 PMC3016282

[9] Silva MMO, Rodrigues MS, Paploski IAD, Kikuti M, Kasper AM, Cruz JS, et al Accuracy of Dengue Reporting by National Surveillance System, Brazil. Emerg Infect Dis J. 2016;22: 336 10.3201/eid2202.150495 26812472 PMC4734515

[10] Kikuti M, Cunha GM, Paploski I a. D, Kasper AM, Silva MMO, Tavares AS, et al Spatial Distribution of Dengue in a Brazilian Urban Slum Setting: Role of Socioeconomic Gradient in Disease Risk. PLoS Negl Trop Dis. 2015;9: e0003937 10.1371/journal.pntd.0003937 26196686 PMC4510880

[11] Lanciotti RS, Calisher CH, Gubler DJ, Chang GJ, Vorndam A V. Rapid detection and typing of dengue viruses from clinical samples by using reverse transcriptase-polymerase chain reaction. J Clin Microbiol. 1992/03/01 ed. 1992;30: 545–551. Available: http://www.ncbi.nlm.nih.gov/pubmed/1372617 1372617 10.1128/jcm.30.3.545-551.1992 PMC265106

[12] Teixeira MDG, Barreto ML, Costa MDCN, Ferreira LD a, Vasconcelos PFC, Cairncross S. Dynamics of dengue virus circulation: A silent epidemic in a complex urban area. Trop Med Int Heal. 2002;7: 757–762. 10.1046/j.1365-3156.2002.00930.x 12225506

[13] Braga C, Luna CF, Martelli CMT, Souza WV De, Cordeiro MT, Alexander N, et al Seroprevalence and risk factors for dengue infection in socio-economically distinct areas of Recife, Brazil. Acta Trop. 2010;113: 234–240. 10.1016/j.actatropica.2009.10.021 19896921 PMC3847853

[14] StataCorp. Stata Statistical Software: Release 14. College Statio, TX: StataCorp LP; 2015.

[15] Peeling RW, Artsob H, Pelegrino JL, Buchy P, Cardosa MJ, Devi S, et al Evaluation of diagnostic tests: dengue. Nat Rev Microbiol. Nature Publishing Group; 2010;8: S30–S37. 10.1038/nrmicro2459 21548185

[16] Andries AC, Duong V, Ngan C, Ong S, Huy R, Sroin KK, et al Field Evaluation and Impact on Clinical Management of a Rapid Diagnostic Kit That Detects Dengue NS1, IgM and IgG. PLoS Negl Trop Dis. 2012;6: e1993 10.1371/journal.pntd.0001993 23301110 PMC3531494

[17] Gan VC, Tan LK, Lye DC, Pok KY, Mok SQ, Chua RCR, et al Diagnosing dengue at the point-of-care: Utility of a rapid combined diagnostic kit in Singapore. PLoS One. 2014;9: 1–6. 10.1371/journal.pone.0090037 24646519 PMC3960091

[18] Vickers IE, Harvey KM, Brown MG, Nelson K, DuCasse MB, Lindo JF. The performance of the SD BIOLINE Dengue DUO rapid immunochromatographic test kit for the detection of NS1 antigen, IgM and IgG antibodies during a dengue type 1 epidemic in Jamaica. J Biomed Sci. 2015;22: 55 10.1186/s12929-015-0164-9 26173484 PMC4502463

[19] Simonnet C, Okandze A, Matheus S, Djossou F, Nacher M, Mahamat A. Prospective evaluation of the SD BIOLINE Dengue Duo rapid test during a dengue virus epidemic. Eur J Clin Microbiol Infect Dis. 2017;36: 2441–2447. 10.1007/s10096-017-3083-8 28831747

[20] Prado PS, Almeida Júnior JTD, Abreu LT de, Silva CG, Souza L da C, Gomes MC, et al Validation and reliability of the rapid diagnostic test “SD Bioeasy Dengue Duo” for dengue diagnosis in Brazil: a phase III study. Mem Inst Oswaldo Cruz. Instituto Oswaldo Cruz, Ministério da Saúde; 2018;113: e170433–e170433. 10.1590/0074-02760170433 29947711 PMC6014722

[21] Carter MJ, Emary KR, Moore CE, Parry CM, Sona S, Putchhat H, et al Rapid Diagnostic Tests for Dengue Virus Infection in Febrile Cambodian Children: Diagnostic Accuracy and Incorporation into Diagnostic Algorithms. PLoS Negl Trop Dis. 2015;9: e0003424 10.1371/journal.pntd.0003424 25710684 PMC4340051

[22] Lertjuthaporn S, Khowawisetsut L, Keawvichit R, Polsrila K, Chuansumrit A, Chokephaibulkit K, et al Identification of changes in dendritic cell subsets that correlate with disease severity in dengue infection. Jin D-Y, editor. PLoS One. 2018;13: e0200564 10.1371/journal.pone.0200564 30001408 PMC6042784

[23] Castañeda DM, Salgado DM, Narváez CF. B cells naturally induced during dengue virus infection release soluble CD27, the plasma level of which is associated with severe forms of pediatric dengue. Virology. 2016;497: 136–145. 10.1016/j.virol.2016.07.014 27467579

[24] Marques RE, Besnard A-G, Maillet I, Fagundes CT, Souza DG, Ryffel B, et al Interleukin-33 contributes to disease severity in Dengue virus infection in mice. Immunology. 2018; 10.1111/imm.12988 30098206 PMC6231004

[25] Piedrahita LD, Agudelo IY, Trujillo AI, Ramírez RE, Osorio JE, Restrepo BN. Evaluation of Commercially Available Assays for Diagnosis of Acute Dengue in Schoolchildren During an Epidemic Period in Medellin, Colombia. Am J Trop Med Hyg. The American Society of Tropical Medicine and Hygiene; 2016;95: 315–321. 10.4269/ajtmh.15-0492 27185768 PMC4973176

[26] Andries AC, Duong V, Ngan C, Ong S, Huy R, Sroin KK, et al Field Evaluation and Impact on Clinical Management of a Rapid Diagnostic Kit That Detects Dengue NS1, IgM and IgG. PLoS Negl Trop Dis. 2012;6: e1993 10.1371/journal.pntd.0001993 23301110 PMC3531494

[27] Hunsperger E a., Yoksan S, Buchy P, Nguyen VC, Sekaran SD, Enria D a., et al Evaluation of commercially available anti-dengue virus immunoglobulin M tests. Emerg Infect Dis. 2009;15: 436–440. 10.3201/eid1503.080923 19239758 PMC2681117

[28] Sánchez-Vargas L a., Sánchez-Marce EE, Vivanco-Cid H. Evaluation of the SD BIOLINE dengue duo rapid test in the course of acute and convalescent dengue infections in a Mexican endemic region. Diagn Microbiol Infect Dis. Elsevier Inc.; 2014;78: 368–372. 10.1016/j.diagmicrobio.2013.12.019 24480246

[29] Guzman MG, Halstead SB, Artsob H, Buchy P, Farrar J, Gubler DJ, et al Dengue: a continuing global threat. Nat Rev Microbiol. 2010;8: S7–S16. 10.1038/nrmicro2460 21079655 PMC4333201

[30] Byrt T. How Good Is That Agreement?. Epidemiology. 1996;7 Available: http://journals.lww.com/epidem/Fulltext/1996/09000/How_Good_Is_That_Agreement__.30.aspx 10.1097/00001648-199609000-00030 8862998

[31] Paploski IAD, Souza RL, Tauro LB, Cardoso CW, Mugabe VA, Alves ABPS, et al EPizootic outbreak of yellow fever virus and risk for human disease in salvador, brazil. Ann Intern Med. 2018;168: 301–302. Available: 10.7326/M17-1949 29114780

[32] Paploski IAD, Prates APPB, Cardoso CW, Kikuti M, Silva MMO, Waller LA, et al Time Lags between Exanthematous Illness Attributed to Zika Virus, Guillain-Barré Syndrome, and Microcephaly, Salvador, Brazil. Emerg Infect Dis J. 2016;22 10.3201/eid2208.160496 27144515 PMC4982160

[33] Faria NR, Azevedo R d. S d. S, Kraemer MUG, Souza R, Cunha MS, Hill SC, et al Zika virus in the Americas: Early epidemiological and genetic findings. Science (80-). 2016;352: 345–349. 10.1126/science.aaf5036 27013429 PMC4918795

[34] Passos SRL, Santos MAB dos, Cerbino-Neto J, Buonora SN, Souza TML, Oliveira RVC de, et al Detection of Zika Virus in April 2013 Patient Samples, Rio de Janeiro, Brazil. Emerg Infect Dis J. 2017;23: 2120 10.3201/eid2312.171375 28953451 PMC5708232

